# Menstrual‐Related Fluctuations in a Juvenile‐Onset Parkinson's Disease Patient Treated with STN‐DBS: Correlation with Local Field Potentials

**DOI:** 10.1002/mdc3.13931

**Published:** 2023-12-06

**Authors:** Elena Contaldi, Gaetano Leogrande, Riccardo Fornaro, Cristoforo Comi, Luca Magistrelli

**Affiliations:** ^1^ Movement Disorders Centre, Neurology Unit, Department of Translational Medicine University of Piemonte Orientale Novara Italy; ^2^ Medtronic Bakken Research Center BV Maastricht The Netherlands; ^3^ Department of Neurosurgery University Hospital “Maggiore Della Carità” Novara Italy; ^4^ Neurology Unit, S. Andrea Hospital, Department of Translational Medicine University of Piemonte Orientale Vercelli Italy; ^5^ Present address: Parkinson Institute ASST G. Pini‐CTO, Via Emilio Bignami 1 20126 Milan Italy

**Keywords:** local field potentials, sex hormones, deep brain stimulation, Parkinson's disease, parkin

Subthalamic nucleus (STN) local field potentials (LFPs) represent useful markers of motor symptoms in Parkinson's disease (PD). Previous evidence reported disease‐specific increased oscillatory activity in the beta frequency band (13–30 Hz) and beta power amplitude correlates with parkinsonian motor impairment.^1^ Novel technologies have been developed to both modulate brain activity and record LFPs. Percept™ PC (Medtronic PLC, MN, USA) is the first commercially available implantable pulse generator (IPG) capable of sensing brain signals while delivering at the same time continuous stimulation. In this study, we exploited the Percept™ PC IPG to better understand the fluctuation of motor symptoms during different phases of the menstrual cycle in a young PD patient treated with STN‐DBS. Indeed, severe menstrual cycle–related reduced responsiveness to medication has been anecdotally reported,^2^ but previous attempts to find correlations between motor signs and hormonal levels have failed,^3^ leaving the mechanism by which sex hormones influence the dopamine system still unclear.

This 21‐year‐old woman, harboring a homozygous mutation c.G859A in the *Parkin* gene, was treated in November 2020 with STN‐DBS (Medtronic® 3389 leads with Percept™ PC IPG) at age 20.^4^ The placement of DBS leads within the STN was confirmed using Lead‐DBS‐v2 software.^5^ For chronic stimulation the following parameters were set: left hemisphere (LeH), case +, contact #2‐, 2.6 mA/60 μs/130 Hz; right hemisphere (RH), case +, contact #9‐, 2.9 mA/60 μs/130 Hz.

The patient showed meaningful clinical benefit after surgery but after a while reported during the luteal phase of her menstrual cycle more “off” periods with painful dystonia. Therefore, in 2022, a study with weekly examinations was started, for a month. Serum levels of estradiol (E_2_), progesterone (P_4_), follicle‐stimulating hormone (FSH), and luteinizing hormone (LH) were consecutively obtained at V0 (first day of the menstrual cycle/early follicular phase), V1 (late follicular phase), V2 (ovulatory phase), and V3 (mid‐luteal phase). Menstrual subphase identification was made based on blood sex hormone measurements and the self‐report of onset of menses, according to previously published recommendations.^6^ Objective evaluation was performed at each time point using the MDS‐UPDRSIII by a movement disorder specialist blinded to the patient's hormonal condition. Concomitant antiparkinsonian therapy (melevodopa/carbidopa 100/25 mg/day) was kept constant throughout the study. The examination was conducted after an overnight withdrawal from dopaminergic medication as follows: stimulation on (stim‐on)/medication off (med‐off), stim‐off/med‐off, and stim‐on/med‐on. Stimulation was discontinued for at least 60 minutes for the stim‐off/med‐off assessment to ensure the complete reappearance of rigidity and bradykinesia.^7^ Stim‐off/med‐on condition was not tested to avoid painful sensations in the off state. Clinical benefit in stim‐on/med‐off and stim‐on/med‐on was calculated as the percentage of MDS‐UPDRSIII score reduction from stim‐off/med‐off. The sensing system was set up as explained in Supporting Information. LFPs were acquired using the Brainsense™ Realtime streaming for 3 min after each clinical evaluation with no changes in the usual parameters of stimulation. LFP data were then exported for offline analysis in MATLAB (The Mathworks, Nattick, MA, USA) as detailed in Supporting Information. The suppression of beta activity in stim‐on/med‐off and stim‐on/med‐on was expressed as the percentage of amplitude reduction compared with stim‐off/med‐off. PSD analysis at different time points is shown in Fig. 1A.

**Figure 1:**
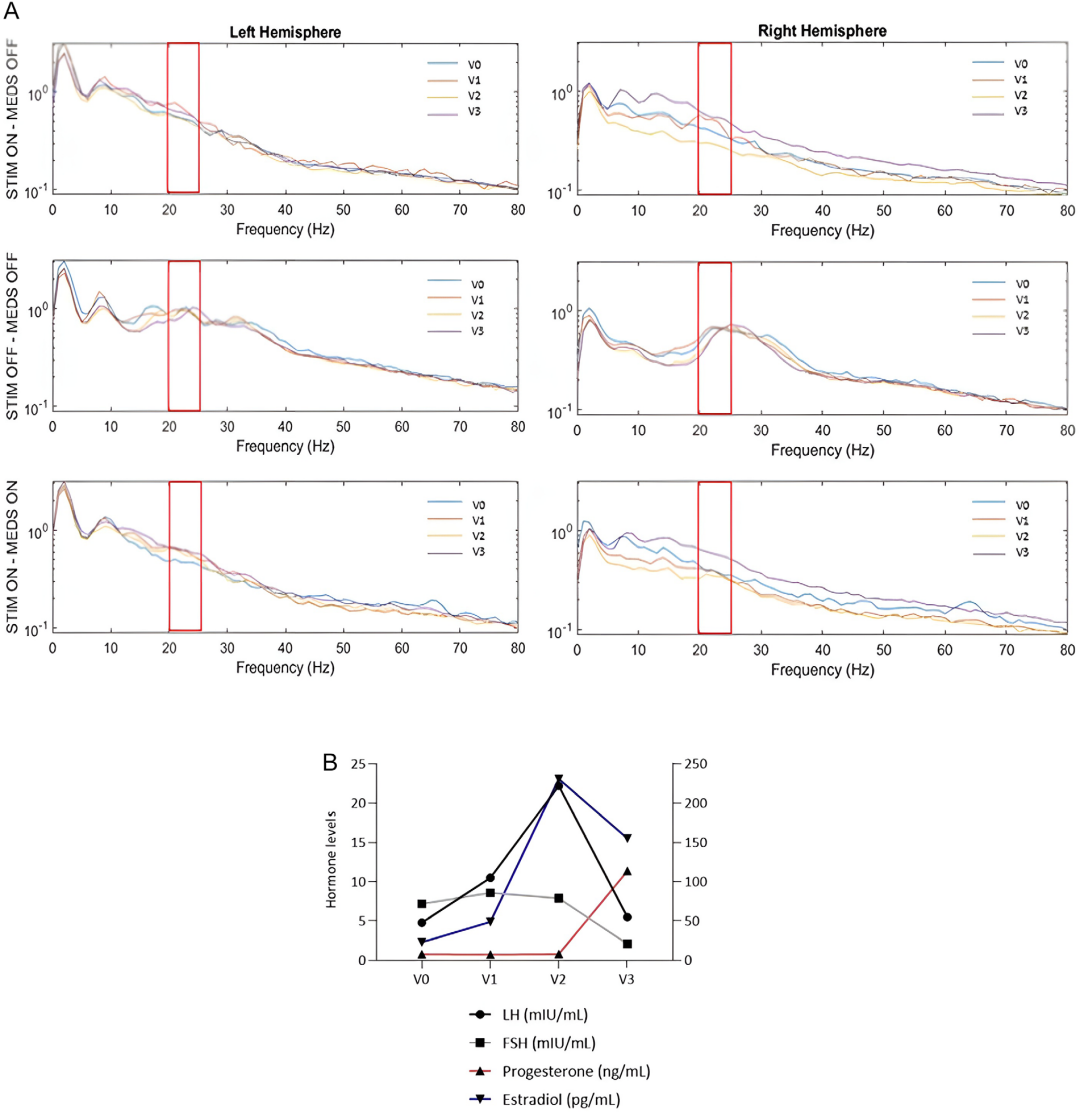
**(Panel A)** Power spectral densities (PSDs, expressed in μV/rtHz) of STN‐LFPs recorded from both hemispheres in all tested experimental conditions at V0 (first day of the menstrual cycle/early follicular phase), V1 (late follicular phase), V2 (ovulatory phase), and V3 (mid‐luteal phase). The beta band frequency of interest is highlighted in red. (**Panel B)** Levels of LH, FSH, Progesterone and Estradiol measured throughout different phases of the menstrual cycle. Levels of LH, FSH and Progesterone are displayed on the left y‐axis whereas levels of Estradiol are displayed on the right y‐axis.

The lowest improvement of motor function was observed at V3 with percentage improvements of MDS‐UPDRSIII scores in stim‐on/med‐off and stim‐on/med‐on of 7% and 34%. Accordingly, the average percentage suppression of beta activity reached its lowest values at V3, respectively, by 17% in stim‐on/med‐off and by 13% in stim‐on/med‐on (Fig. S1.). V3 was characterized by a sharp increase in P_4_ levels, Figure 1B. In an exploratory linear regression analysis (details in Supporting Information), the percentage of beta activity reduction in stim‐on/med‐on was predicted by the levels of P_4_ (β = −0.982, *P* = 0.018), especially in the RH (β = −0.994, *P* = 0.006). Conversely, no significant association was found between sex hormone levels and objective clinical measures. These results were also confirmed when analyzing P_4_ increment as an independent variable. This discrepancy can be explained in several ways: first, tremor and other cardinal motor symptoms (ie, postural instability and gait problems) have been linked to other frequency bands^8^ and thus may affect the correlation with the suppression of beta power; second, the scoring system of MDS‐UPDRSIII is nonlinear, thus limiting the elucidation of the exact relationship with sex hormone levels.

This study provides novel insights into the role of sex hormones in PD‐related motor fluctuations. Available literature has yielded conflicting results so far, reporting both enhancement and suppression of the striatal dopaminergic system by estrogens and progesterone in PD.^9^, ^10^, ^11^

Our case confirms the feasible role of LFP recordings of STN beta activity as biomarkers to track different parkinsonian conditions. Additionally, several intriguing questions regarding the complex relationship between sex hormones and motor fluctuations may arise. In a previous study,^3^ although PD severity changed during different menstrual phases, there was no significant correlation between objective measures of parkinsonism and hormone levels. In the present case report, however, we found novel associations between P_4_ levels and the modulation of beta band activity in the STN. Interestingly, beta band modulation is influenced by P_4_ in stim‐on/med‐on condition and not stim‐on/med‐off, thus emphasizing the role of sex hormones on the response to dopaminergic medication. Furthermore, our study explores neurophysiological features in a specific genetic subtype of PD: recent works^12^, ^13^ have reported conflicting results regarding the role of genetic etiology in STN physiology. Whether the present findings can be explained by the patient's genetic status, the direct effect of sex hormones on the nigrostriatal pathway through the modulation of dopamine receptor availability, or the interaction between E_2_ and P_4_, is still to be clarified. We acknowledge this study has several limitations: (i) since this investigation is based on a single patient, our findings should be taken cautiously and further assessed in the context of larger studies; (ii) stim‐off/med‐on condition was not evaluated; (iii) even though stim‐off/med‐off assessment was conducted in line with previous research^7^ we cannot exclude that some parkinsonian symptoms would have required longer discontinuation of stimulation to reappear.^14^

Despite these limitations, we believe that a deeper understanding of such physiological signatures and their modulation by different exogenous and endogenous factors will be of paramount importance to inform targeted strategies in the expanding scenario of adaptive DBS paradigms.

## Author Roles

(1) Research Project: A. Conception, B. Organization, C. Execution; (2) Statistical Analysis: A. Design, B. Execution, C. Review and Critique; (3) Manuscript Preparation: A. Writing of the first draft, B. Review and Critique.

E.C.: 1A, 1B, 1C, 2A, 2B, 3A

G.L.: 1A, 1B, 1C, 2C, 3A

R.F.: 1C, 2C, 3B

C.C.: 1C, 2C, 3B

L.M.: 1B, 1C, 2C, 3B.

## Disclosures

**Ethical Compliance Statement:** The study was performed following the ethical standards as laid down in the 1964 Declaration of Helsinki and following the international research ethical principles involving human subjects. The patient was enrolled in the context of a study aiming at the characterization of biomarkers in genetic forms of PD and the institutional Ethics Committee of Novara University Hospital “Maggiore della Carità” approved the study protocol (CE 149/2022). Written informed consent was obtained from the patient.

**Funding Sources and Conflicts of Interest:** No specific funding was received for conducting this study. GL is a Medtronic employee, the other authors have no conflicts of interest to declare.

**Financial Disclosures for the Previous 12 Months:** The authors declare that there are no additional disclosures to report.

## Supporting information


**Supplementary Figure S1.** Percentage of clinical improvement and STN beta peak reduction in stim‐on/med‐off and stim‐on/med‐on from stim‐off/med‐off condition at different time points.
