# Supplementary material for: Menstrual‐Related Fluctuations in a Juvenile‐Onset Parkinson's Disease Patient Treated with STN‐DBS: Correlation with Local Field Potentials
Source: Mov Disord Clin Pract. 2023 Dec 6;11(1):101–4. doi: 10.1002/mdc3.13931 (PMC10828624; doi:10.1002/mdc3.13931)
Supplement: Supplementary file 1 — Supplementary Figure S1. Percentage of clinical improvement and STN beta peak reduction in stim‐on/med‐off and stim‐on/med‐on from stim‐off/med‐off condition at different time points. [file MDC3-11-101-s001.docx]

**Supplementary Materials**

**Sensing system setup**

The sensing system was set up to record LFP differentially between contacts #1 and #3 and with center frequency 19.53 Hz on the LeH, and between contacts #8 and #10 and with center frequency 21.48 Hz on the RH. Center frequencies were selected based on the peaks observed in the spectra of the LFP that had amplitude of 3.36 μVp and 1.49 μVp on the LeH and RH, respectively.

**LFP data analysis**

Raw data were assessed visually and portions of the recordings with artifacts were excluded from the signals in both hemispheres. The LFP power spectral density (PSD) of the remaining portions of the LFP raw data in the time domain was then estimated with Welch’s method (1sec Hamming window, 60% overlap, 250 points). The amount of power in the band 20-25 Hz, selected empirically based on preliminary results, was calculated by summing the squared values of the components of the PSD within the band.

**Statistical analysis**

Simple linear regression analysis was performed to investigate the association between LFP/clinical measures and sex hormone levels. More in detail, two sets of models were analyzed: 1) the percentage of beta activity reduction (stim-on/med-on and stim-on/med-off compared with stim-off/med-off) was used as the dependent variable, and levels of sex hormones (E_2_, P_4_, FSH, LH) as sequential independent variables; 2) the percentage improvement of MDS-UPDRSIII scores (stim-on/med-on and stim-on/med-off compared with stim-off/med-off) was used as the dependent variable with the same group of independent variables. Analyses were performed with SPSS version 25 (IBM Corporation, Armonk, USA).

**Supplementary Figure S1**
